# Photobiomodulation Improves Anti-Tumor Efficacy of Photodynamic Therapy against Resistant MCF-7 Cancer Cells

**DOI:** 10.3390/biomedicines11061547

**Published:** 2023-05-26

**Authors:** Eric Chekwube Aniogo, Blassan P. George, Heidi Abrahamse

**Affiliations:** Laser Research Centre, Faculty of Health Sciences, University of Johannesburg, P.O. Box 17011, Doornfontein 2028, South Africa; ericaniogo@gmail.com (E.C.A.); blassang@uj.ac.za (B.P.G.)

**Keywords:** apoptosis, autophagy, resistant cancer cells, photobiomodulation, photodynamic therapy

## Abstract

Cancer resistance is a primary concern in cancer treatment, and developing an effective modality or strategy to improve therapeutic outcomes is imperative. Photodynamic therapy (PDT) is a treatment modality that targets the tumor with a photoactive molecule and light for the specific destruction of cancer cells. Photobiomodulation (PBM) is a light exposure of cells to energize their biomolecules to respond to therapy. In the present study, we used PBM to mediate and improve the anti-tumor efficacy of zinc phthalocyanine tetrasulfonic acid (ZnPcS4)-PDT on resistant MCF-7 breast cancer cells and explore molecular changes associated with cell death. Different laser irradiation models were used for PBM and PDT combination. The combined treatment demonstrated an additive effect on the viability and Annexin-V/PI-staining cell death assessed through MTT assay and mitochondrial release of cytochrome c. Rhodamine (Rh123) showed increased affinity to mitochondrial disruption of the strategic treatment with PBM and PDT. Results from the autophagy assay indicate an interplay between the mitochondrial and autophagic proteins. These findings were indicative that PBM might improve the anti-tumor of PDT by inducing autophagy in resistant MCF-7 breast cancer cells that evade apoptosis.

## 1. Introduction

Cancer drug resistance holds a significant threat to the treatment and control of cancer. It is a major setback that many experts in the field are racing to conquer by integrating a combination of different treatment strategies to arrest tumor relapse and curb its menaces [1,2]. The application of light in therapy has been used for many years for tissue repair, mucositis, and muscle regeneration. This technique modifies the upregulation of endogenous enzyme activity through cell signaling and tissue metabolism to promote proliferation and inflammatory response in cells [3]. It has also been applied in treating wounds and cancer through preferential activation and restimulation of mitochondria [4,5]. Light irradiation, or photobiomodulation, is a non-invasive therapy for many diseases where a preferential light source of the near-infrared wavelength region (600–800 nm) is used to stimulate, heal, and regenerate damaged tissues [3]. The photobiomodulation process activates potential redox changes and upregulates proteins such as mitofusin 2 and cytochrome c oxidase, which modulate antioxidant responses, oxygenation, and tissue repair [6]. PBM has also shown efficacy in accelerating the healing process of oral mucositis in cancer patients. At the cellular level, PBM induces the availability of ATP, resulting in cell proliferation and enhanced collagen synthesis that encourages healing [7]. Through its secondary effects, PBM can cause increased blood flow, antioxidant defense, and alteration of growth factors and cytokine expression patterns [8]. Tsai and colleagues examined the pre-treatment of osteosarcoma (MG-63) cells with low laser light therapy before PDT. This approach increased PDT cell killing through ATP synthesis, potentiating drug uptake, and cell apoptosis [9]. In another study, ruthenium phthalocyanine compound as a photosensitizer was used to induce a synergistic killing with PBM in melanoma cancer [5]. The combination of PBM and PDT showed improved tumor microenvironment modification, allowing apoptosis and promoting anticancer efficacy with less toxic effects on healthy cells [5]. 

Conversely, PDT combines light irradiation, photosensitizer, and oxygen to treat diseases such as cancer by generating toxic amounts of reactive oxygen species (ROS) in the target tissue [10]. PDT is well known in cancer treatment and other anti-microbial applications due to its advantages, including localized killing, tissue sparing, good cosmetic outcomes, and effective tumor eradication [11]. PDT is promising to serve as an adjuvant alternative after surgery in patients with macroscopic margins where resection is impossible [12]. It has been applied for treating cancer resistance repetitively or in combination due to its complex biochemical reactions and multimodal targeting [10,13]. PDT procedure involves the injection of a photosensitizer which is taken up by proliferating malignant cells, followed by its activation with non-thermal light of a specific wavelength to generate oxidative stress and severe vascular damage that leads to cell death through mitochondria-signaling pathways [14]. PDT treatment procedure for premalignant and early malignant lesions has been accepted for use in endo-bronchial, endo-esophageal lesions of the skin (actinic keratosis), bladder, breast, stomach, and oral cavity [15]. 

In this study, we propose PBM as a good alternative for combination therapy to improve the efficacy of PDT on resistant tumor cells. PBM alone is unsuitable for tumor eradication; however, its role in enhancing the susceptibility of tumor cells to chemotherapy has been studied [16,17]. The combination of PDT and chemotherapy has synergistic effects in cancer treatment [18]. A new strategy of combining PBM and PDT might offer improved efficacy on resistant cancer with the impact that PBM will increase cancer susceptibility to therapy. In vitro, a combination of this approach in oral cavity carcinoma showed that PBM increased PS tumor uptake and ROS production with PDT [3]. Pevna and colleagues reported that PBM switches the autophagic response of the human dermal fibroblast to apoptosis when the two therapies are used together, thus improving the treatment efficacy and yielding a better therapeutic outcome, unlike when the individual treatments were used alone [5,19]. Effective PDT depends on ROS photo-production, which is proportional to the amount of oxygen concentration, and the laser wavelength used. The hypothesis mechanism suggests that PBM mediated increased metabolism and ATP production that results in higher PS uptake and energy supply to undergo apoptosis. This depends on the fluence of the light source, PS incubation time, and cell lines used. Hence, because PBM alters cell metabolism and increases ATP synthesis, it will be an excellent combination agent to improve the PDT anti-tumor effect on resistant cancer cells. 

Thus, the present study showed enhanced anti-tumor efficacy of zinc phthalocyanine tetrasulfonic acid (ZnPcS4)-PDT to mediate cell death on resistant MCF-7 breast cancer cells. We also observed that different laser irradiation fluences modulate resistant cancer cell susceptibility to cell death. 

## 2. Materials and Methods

### 2.1. Cell Culture Preparation

The human breast cancer cell line (MCF-7; ATCC HTB-22) induced with suboptimal Doxorubicin (1 µM/mL; Sigma-Aldrich, Johannesburg, South Africa) to express increased P-glycoprotein expression was used for this experiment and referred to as MCF-7/DOX cells. P-glycoprotein expression measured with flow cytometry showed a 4-fold increased expression of MCF-7/DOX cells compared to parental MCF-7 cells [13]. The cells were grown in complete (10%) Fetal Bovine Serum (FBS, 10499-044; Sigma-Aldrich, Johannesburg, South Africa, 1% Penicillin–Streptomycin (P4333; Sigma-Aldrich, Johannesburg, South Africa), and 1% Amphotericin B (A2942; Sigma-Aldrich, Johannesburg, South Africa) Dulbecco’s Modified Eagle’s Medium (DMEM; D5796, Sigma-Aldrich, Johannesburg South Africa) with 1 µM/mL Dox and incubated at 37 °C and 5% CO_2_. Experimental cells of 5 × 10^5^ cells/cm^2^ were seeded in 35 mm culture dishes and allowed 4 h for attachment before treatment. 

### 2.2. Photobiomodulation and Photodynamic Treatment

**Figure 1:**
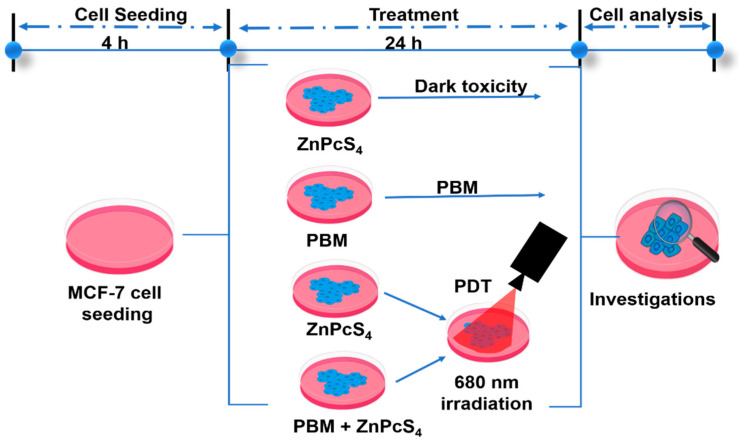
The schematic representation of the experimental procedure.

The photosensitizer zinc tetrasulfonic acid phthalocyanine (ZnPcS4) used in this study was purchased from Santa Cruz^®^ Biotechnology (sc-264509A, Anatech (Pty) Ltd., Johannesburg, South Africa). It absorbs light at 674 nm and hence, a 681 nm diode laser beam was used for photobiomodulation and photodynamic therapy laser irradiation. The laser parameters used for the experiment are summarized in Table 1 and the scheme of the experimental procedure is shown in Figure 1. The laser was provided and set up by the Council for Scientific and Industrial Research (CSIR)/National Laser Centre (NLC) (Pretoria, South Africa). The cells were grouped into dark, PBM, PDT and PBM + PDT. Reports have shown that a 45 µM concentration of ZnPcS4 at 20 J/cm^2^ irradiation yielded 50% inhibitory concentration [13]. Hence, we use a suboptimal 20 µM concentration of ZnPcS4 across different fluencies of laser light in the dark, laser irradiation alone, PDT, and a combination of laser irradiation (PBM) and PDT. For dark control, 20 µM of ZnPcS4 was added to MCF-7/DOX cells and incubated at 37 °C overnight; for PBM, cells were exposed to laser irradiation alone; for PDT, ZnPcS4 was added and allowed to internalize for 4 h before irradiation, and for the PBM + PDT group, cells were irradiated first before ZnPcS4 was added and incubated for 4 h followed by second irradiation. The cells were washed after treatment with phosphate-buffered saline (PBS), added to new completed media, and put back in the incubator for 20 h before cellular response assessment. 

### 2.3. MTT Assay

The MCF-7/DOX cells in exponential growth were harvested, and the effect of the treatment regime was evaluated using a 3-(4,5-dimethylthiazol-2-yl)-2,5-diphenyltetrazolium bromide (MTT) (11465007001; Roche, Johannesburg, South Africa) colorimetric assay for viability, which is based on the reduction of MTT tetrazole salt to purple formazan. Thus, the measurement of the formazan dye produced is proportionally related to the cell’s viability. 

### 2.4. Staining and Measurement of Mitochondrial Integrity with Rhodamine 123 (Rh-123)

Rh-123 (R8004; Sigma-Aldrich, Johannesburg, South Africa) is a fluorescent dye that selectively accumulates in the mitochondria and is used to monitor the membrane integrity of the cells after treatment. Briefly, the cells were resuspended in 20 µg/mL of Rh-123 in PBS and incubated for 30 min at 37 °C. After a 30-min incubation, the cells were washed 3 times with PBS and lysed with lysis buffer. This was followed by fluorescence measurement at excitation/508 nm and emission/525 nm wavelength for the amount of Rh-123 retained in the mitochondria using a Perkin-Elmer, Victor Nino^TM^ multimode plate (Midrand, South Africa) reader. 

### 2.5. ELISA Assay for Cytochrome c Protein Detection

Mitochondrial cytochrome c release was measured using ELISA (KH01051, ThermoFisher Scientific, Johannesburg, South Africa). This measurement method captured target antigens and detected antibodies immobilized on the 96-well plate. Hence, an equal volume (100 µL) of samples and biotin conjugate solution were added and rocked on a shaker during a 1 h incubation at room temperature. Cells were washed with 350 µL of 1× wash buffer, dried on a paper towel, and incubated at 100 µL of 1× streptavidin-horseradish peroxidase (HRP) solution for 30 min. The HRP solution was washed off with 1× wash solution and incubated with 100 µL of stabilized chromogen for 30 min in the dark. After that, the stop solution of 100 µL was added and shaken for 1 min. The optical density reading was measured at 450 nm (Perkin-Elmer, Victor Nino^TM^ multimode plate reader, Midrand, South Africa).

### 2.6. Annexin V/Propidium Iodide (PI) Flow Cytometric Analysis of Cell Death

Fluorescein Isothiocyanate (FITC) Annexin V/PI Apoptosis detection kit II (BD Pharmingen^TM^: 556570, the Scientific Group, Roodeport, South Africa) was used to detect apoptosis in treated MCF-7/DOX cells. After dark, PBM, PDT, and combination-treatment cells were harvested, washed twice, and stained with equal volume (5 µL) of FITC Annexin V and PI solution in 100 µL of a kit binding resolution. The mixture was incubated in the dark at room temperature for 15 min and analyzed using a BD Accuri™ C6 flow cytometer (BD Biosciences, the Scientific Group, Roodeport, South Africa).

### 2.7. Fluorescence Measurement for Autophagy 

The fluorescence autophagy kit (MAK 138, Sigma-Aldrich, Johannesburg, South Africa) method measures the cell degradation process by measuring the fluorescence autophagosome marker. Briefly, the cell pellet in culture media was plated at 2 × 10^4^ cells/100 µL per well in black poly-d-lysine 96 plates. After 2 h to attain the hemostatic recovery, the media was removed, and 100 µL of autophagosome diluted detection reagents were added to each well and incubated at 37 °C with 5% CO_2_ for 1 h. Cells were washed with wash buffer 3 times and resuspended in 100 µL of PBS. Fluorescent intensity was determined using the relative fluorescence unit (RFU), which represented the autophagosome marker. It was measured using excitation 360 nm/emission 520 nm. 

### 2.8. Statistical Analysis

The experiments were duplicated for at least three repeats (n = 3). The dark untreated control cells were used to compare the treated cells in one-way ANOVA statistical analysis (Dunnett test). Data represented mean ± standard error of the mean (SEM) and was analyzed using GraphPad Prism 7.0 (GraphPad by Dotmatics, Boston, MA, USA); *p*-values less than 0.05 (*), 0.01 (**), and 0.001 (***) were used as statistical differences.

## 3. Results

### 3.1. Measurement of Cellular Viability

**Figure 2:**
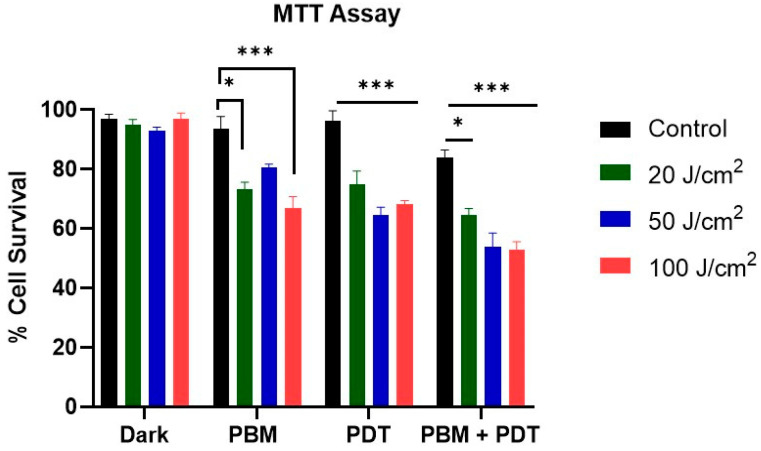
Represents the MTT cell viability assay of MCF-7/DOX cells. This assay showed a progressive decrease as laser irradiation increased. Cell viability decreased significantly in PDT and combination-treatment groups compared to dark untreated controls. Significant differences were presented as * *p* < 0.05, and *** *p* < 0.001.

We examined the effect of various treatment conditions on the viability of MCF-7/DOX cells. MTT results revealed a significant decrease in the viability of the PDT and PBM + PDT combination-treated groups. Additionally, it was observed that as the laser irradiation doses increase, so the cellular viability decreases. Dark control groups showed no difference from the untreated group (Figure 2).

### 3.2. Assessment of Mitochondria Integrity with Rhodamine 123 Staining

**Figure 3:**
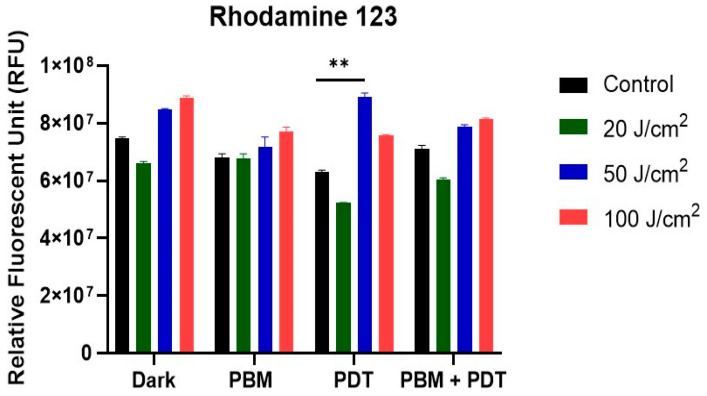
Rhodamine 123 measurements of the mitochondria membrane integrity of MCF-7/DOX cells with different treatment groups. Increased accumulation of Rh-123 occurred in PDT and combination-treated groups with 50 J/cm^2^. Significant differences were represented as ** *p* < 0.01.

Rh-123 has a high affinity for mitochondria, dependent on the negatively charged membrane potential across the inner mitochondrial membrane. This property was used as an index to measure the integrity of the mitochondria after treating the resistant cell lines with different degrees of laser irradiation. Results showed an increased accumulation of Rh-123 in the PDT and combination-treated groups (Figure 3).

### 3.3. ELISA Measurement of Cytochrome c Proteins Release

**Figure 4:**
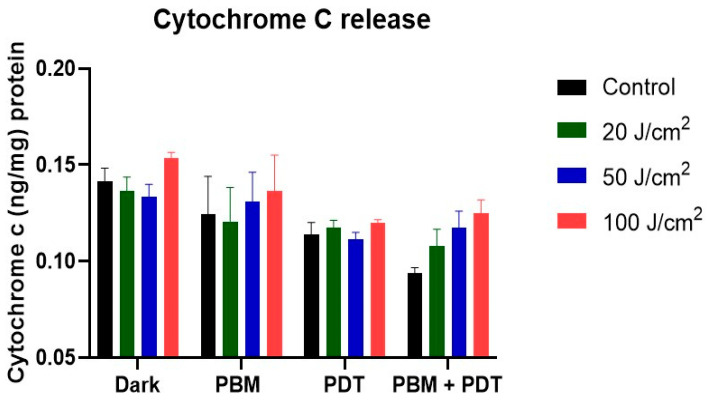
The enzyme immunoassay measurement of cytochrome c protein release. Results showed an increased pattern of cytochrome c release in different treatment groups and irradiation conditions used.

The photodamage of the mitochondria was further assessed with cytochrome c apoptotic protein release using an enzyme-linked immunosorbent assay. The results showed increased release of cytochrome c in PDT and combination-treatment groups. This release follows a pattern of progressive increase in both 50 and 100 J/cm^2^ (Figure 4). This result conforms with the Rh-123 measurement of mitochondrial integrity and shows that PDT and its combination with PBM might impact mitochondria.

### 3.4. Annexin V/PI Staining

**Figure 5:**
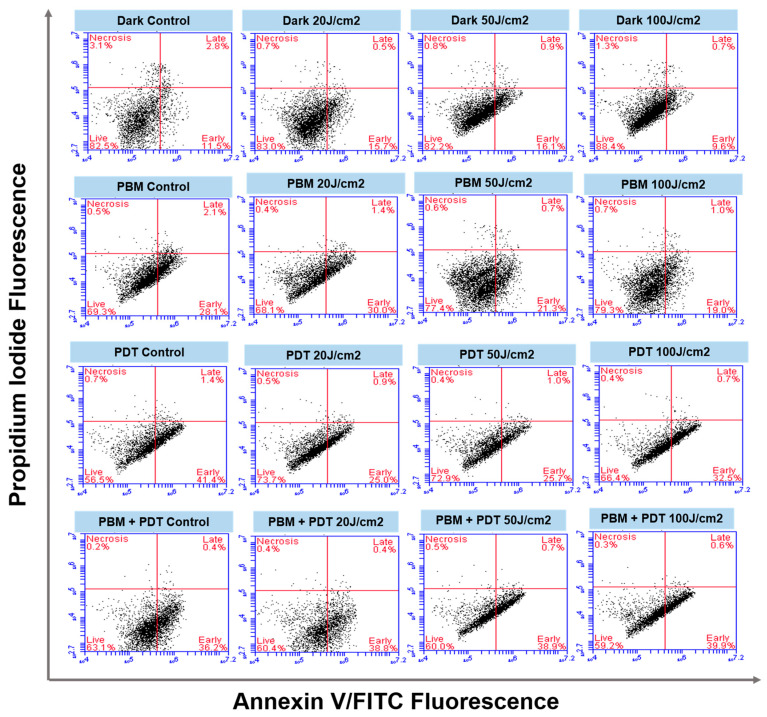
Quadrant distribution of MCF-7/DOX cells in different phases of apoptosis. The cells were stained with Annexin V/FITC and showed live, early, and late apoptotic cells. The untreated cells were used as control, and different treatment groups (Dark, PBM, PDT, and PBM + PDT) for 20, 50, and 100 J/cm^2^ were labeled, respectively.

The flow cytometry results showed the time-dependent process of apoptosis of MCF-7/DOX cells in different quadrants (Figure 5). Various treatment groups (dark, PBM, PDT, and combination) showed the initiation of apoptotic response dependent on the laser fluencies applied. The high laser irradiation of 50 and 100 J/cm^2^ increased the number of cells entering the early apoptotic phase compared to the untreated dark control.

### 3.5. Analysis of Autophagy

**Figure 6:**
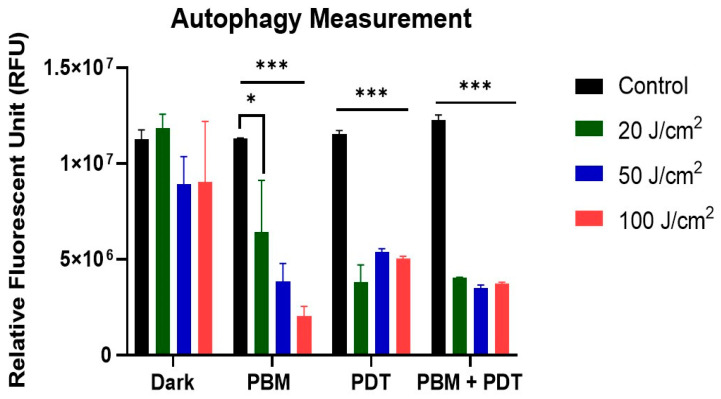
Fluorescence measurement of autophagy. The untreated dark control showed no difference, but other treatment groups (PBM, PDT, and PBM + PDT) were significant at different laser irradiations (20, 50, and 100 J/cm^2^). Significant differences were presented as * *p* < 0.05, and *** *p* < 0.001.

The degradation process of autophagy was measured with fluorescence analysis of the autophagosome marker according to the instructions in the assay kit (MAK 138, Sigma-Aldrich, Johannesburg, South Africa). This assay revealed the effect of different treatment groups on MCF-7/DOX cells. Both PBM and PDT, when used alone or in combination, induced the release of autophagosome marker, which was significant at different laser irradiations (20, 50, and 100 J/cm^2^). The dark untreated controls were insignificant, indicating the photoactivation of autophagic response. The results in the PBM group showed a different degree of autophagy induction by additional laser irradiation. However, in the combination-treatment group, the irradiation dose-dependent response to autophagy was normalized to almost the same effect (Figure 6).

## 4. Discussion

Cancer resistance has emerged as a threat because of the dynamic challenges of multidrug resistance, resulting from increased expression of P-gp that prevents and extrudes drugs from the cell [20,21,22,23]. Drug resistance stems from intrinsic and acquired mechanisms that often require a combination of therapies [10]. Studies using PDT in combination with chemotherapy showed an increased killing effect on drug-resistant cancer cells [24]. Yang and colleagues in their combination study with PDT on resistant MCF-7/ADR cells found that low-dose PDT facilitates the endocytosis of drug carrier nanostructure loaded with chemotherapeutic drug and photosensitizer. Subsequent Irradiation of these cells showed a significant cytotoxicity through the synergistic effect of PDT and chemotherapy [24]. In the present study, we used PBM to mediate and improve the anti-tumor efficacy of zinc phthalocyanine tetrasulfonic acid (ZnPcS4)-PDT on resistant MCF-7 breast cancer cell lines. The MCF-7/DOX cells used in the study were induced to resist Doxorubicin (DOX) chemotherapy through repeated cycles of Dox exposure [13]. PDT with Zinc phthalocyanine tetrasulfonic acid mediated has shown tumoricidal effects on wide-type MCF-7 and its resistant sublines cells [13]. However, the impact on resistant sublines was less, prompting us to hypothesize that a combination of PBM and PDT can increase the susceptibility of MCF-7/DOX cells to cell death. Several reports have suggested evidence of PBM modulation of many intracellular processes and gene transcription changes related to the extracellular matrix, calcium homeostasis, stress signaling, and apoptosis [25]. At the same time, others proposed that PBM upregulates mitochondrial respiratory chain enzyme through light activation of cytochrome c oxidase related to cells’ response to PDT [26]. PBM stimulates and regenerates damaged tissues and has been less-often described as a possible mechanism to enhance cancer treatment. Bensadoun and colleagues [27] reported that PBM might affect pathways linked to harmful tumor cell proliferation and antiapoptotic effects. The research further highlighted that those malignant cells respond differently from PBM, depending on the parameters used. In another study, human breast and melanoma cells were used to investigate the effect of diverse doses of PBM, and their results reduced the impact on cell proliferation [28]. The metabolic activity assay of MTT was used to determine cellular photodamage among the various treatment groups (PBM, PDT, and combination). The result revealed a significant difference in cell viability of both the PDT and PBM + PDT combination-treated groups. PBM also had an additive effect in the reduced cell viability observed. We also discovered that as laser irradiation doses increase, cellular viability decreases (Figure 2).

The rate of cell death was studied using Annexin V/PI staining, which detects phosphatidylserine, an apoptotic indicator, with flow cytometry. Ideally, apoptosis is the targeted endpoint of most cancer therapy, but due to resistance cancer relapses after treatment [29]. Phosphatidylserine is a protein expressed by cells undergoing apoptosis that has an affinity to annexin V. Results from this staining showed many cells stained positive for annexin V after PBM + PDT, indicating apoptosis. The percentage rate of positive cells in the combination group differed from those observed with dark control and PBM alone under the same conditions (Figure 5). Though it is difficult to accurately determine the combination experiment efficacy rate based on this measurement, nearly 40% of cells were in the early apoptotic phase in the PBM + PDT group. This might result from the oxidative stress of costimulatory and killing responses related to increased production of reactive oxygen species. Apoptosis in PDT-treated cells depends on factors such as intracellular localization of the sensitizers [30], light doses of different irradiation [31,32], and the specific properties of the target cell [33,34]. Comparison of cell death after PDT in various cancer cell lines (lungs, oral squamous, esophageal, bladder, and cervical carcinoma) showed phototoxic effects characterized by loss of mitochondrial membrane, increased ROS generation, and lipid peroxidation [35].

The mitochondrial membrane integrity measured with rhodamine 123 showed increased accumulation in the combination-treatment group. This suggests increased destabilization and generation of oxidative stress by PBM + PDT. Rh-123 is a substrate for P-gp and has been reported as a principal P-gp activity in the efflux mechanism of cancer cells [36]. In addition, the photodamage of the mitochondria was prominent in the cytochrome c measurement assay conducted. In the PBM + PDT group, an increased number of proteins were released, which suggests triggers of apoptosis and damage.

The process of apoptosis can be initiated via intrinsic (or mitochondrial) damage through the release of cytochrome c [37]. Mitochondria are vital in controlling the release of essential factors in the apoptotic cascade. Thus, cytochrome c release is usually accomplished through the opening of the mitochondrial membrane permeability transition pores, which activates the killer protease family proteins called the caspases through the complex formation of apoptotic-protease activating factor-1 [15]. Our results showed that the amount of cytochrome c release in the PBM + PDT group was insignificant, which suggests minimal photodamage; that suffices to say that the photodamaging effect of PDT was remedied with photobiomodulation. Pevna et al. [19] reported that PBM induced autophagy in human dermal fibroblast cells and hypothesized that appropriate regulation of PBM could help cells escape photodestruction and improve treatment efficacy. PDT-mediated hypericin has been reported to remarkably delay the release of cytochrome c in Bcl-2 overexpressing cells due to less mitochondrial membrane potential damage [38]. However, our research found photodamage of the mitochondria and postulated that resistant cells treated with PBM and PDT were eliminated by autophagy, which is the degradation or repair process of organelle damage. Reports have stated that low-dose PDT can stimulate the reverse of apoptosis through autophagy, whereas high-dose PDT associates both cell death mechanisms [35]. The measurement of autophagy was then investigated, and the result showed an increased release of autophagosome markers in the PDT and PBM + PDT groups. Oxidative stress balance is crucial in determining cell fate, whether to induce apoptosis or autophagy. The observations made from these experiments are the basis on which we suggest that PBM might be sole in enhancing the efficacy of PDT by causing mitochondrial imbalance and continuous release of cytochrome c, which triggers the generation of reactive oxygen species and cellular apoptosis.

## 5. Conclusions

In conclusion, autophagy and apoptosis are two interesting cellular death-signaling pathways regulated by PBM and PDT in cancer cells. We have proved that these pathways are stimulated in PBM + PDT-treated groups. PBM upregulated the formation and detection of autophagosome markers, and combining it with PDT could help induce more photodamage and improve treatment chances. The stimulating effect of PBM shown here might differ in vivo depending on many factors associated with the tumor microenvironment, which warrants further studies.

## Figures and Tables

**Table 1:** Parameters of 681 nm diode laser used.

Parameter	
Manufacturer	Optoelectronics. Tech. Co. LTD
Model no.	PSU—III—LED (MRL: 680–800 mW)
Wavelength	681 nm
Wave emission	Continuous
Spot size	9.1 cm^2^
Power output	194 ± 5 mW
Fluences	20, 50, 100 J/cm^2^
Irradiation time	14 min ± 32 s

## Data Availability

Data available on request.
